# Emerging biologic frontiers for Sjogren’s syndrome: Unveiling novel approaches with emphasis on extra glandular pathology

**DOI:** 10.3389/fphar.2024.1377055

**Published:** 2024-05-17

**Authors:** Xiao Xiao Li, Maierhaba Maitiyaer, Qing Tan, Wen Hui Huang, Yu Liu, Zhi Ping Liu, Yue Qiang Wen, Yu Zheng, Xing Chen, Rui Lin Chen, Yi Tao, Shui Lian Yu

**Affiliations:** ^1^ Department of Rheumatology, The Second Affiliated Hospital of Guangzhou Medical University, Guangzhou Medical University, Guangzhou, China; ^2^ Department of Clinical Medicine, The First Clinical Medical School of Guangzhou Medical University, Guangzhou Medical University, Guangzhou, China; ^3^ Ophthalmic Center, the Second Affiliated Hospital of Guangzhou Medical University, Guangzhou, China; ^4^ Department of Nephrology, The Second Affiliated Hospital of Guangzhou Medical University, Guangzhou Medical University, Guangzhou, China; ^5^ Department of Urology, The Second Affiliated Hospital of Guangzhou Medical University, Guangzhou Medical University, Guangzhou, China; ^6^ Department of Geriatrics, The Second Affiliated Hospital of Guangzhou Medical University, Guangzhou Medical University, Guangzhou, China

**Keywords:** Sjögren’s syndrome, extraglandular symptom, biologic therapies, primary Sjögren’s syndrome, cytokines and chemokines

## Abstract

Primary Sjögren’s Syndrome (pSS) is a complex autoimmune disorder characterized by exocrine gland dysfunction, leading to dry eyes and mouth. Despite growing interest in biologic therapies for pSS, FDA approval has proven challenging due to trial complications. This review addresses the absence of a molecular-target-based approach to biologic therapy development and highlights novel research on drug targets and clinical trials. A literature search identified potential pSS treatment targets and recent advances in molecular understanding. Overlooking extraglandular symptoms like fatigue and depression is a notable gap in trials. Emerging biologic agents targeting cytokines, signal pathways, and immune responses have proven efficacy. These novel therapies could complement existing methods for symptom alleviation. Improved grading systems accounting for extraglandular symptoms are needed. The future of pSS treatment may involve gene, stem-cell, and tissue-engineering therapies. This narrative review offers insights into advancing pSS management through innovative biologic interventions.

## 1 Introduction

Sjögren’s Syndrome (SS) is a complex chronic autoimmune disorder, occurring in both primary and secondary forms. The manifestations of SS can be broadly classified into non-specific, peri-epithelial (encompassing both glandular and extraglandular areas), extra-epithelial (inherently extraglandular), and lymphoma-related types. Typically, SS is characterized by diminished function of the salivary and lacrimal glands (Pertovaara et al., 1999), leading to symptoms such as dry eyes and dry mouth (Mariette and Criswell, 2018). Despite the prominent glandular symptoms, SS also involves a broader range of extranglandular features, characterized by diverse symptoms affecting both visceral and non-visceral systems. Visceral manifestations involve the pulmonary, cardiac, renal, gastrointestinal, endocrine, central nervous, and peripheral nervous systems. Concurrently, non-visceral manifestations predominantly present in the musculoskeletal and cutaneous systems (Fox, 2005). A growing body of research indicates that, in evaluating patients with SS, individuals presenting with extraglandular symptoms such as fatigue, depression, and anxiety tend to experience a lower overall quality of life compared to their counterparts (Kotsis et al., 2014; Miglianico et al., 2022). A study of 639 SS patients found that 49.5% showed symptoms of depression and anxiety, significantly higher than the general population’s 15.7% prevalaence. Concurrently, an evaluation of the health-related quality of life for these patients highlighted pain and depression as the two predominant factors significantly impacting the life quality assessment in individuals with SS (Lendrem et al., 2014). Additionally, these patients often face poorer long-term outcomes, including an increased likelihood of complications.

**FIGURE 1:**
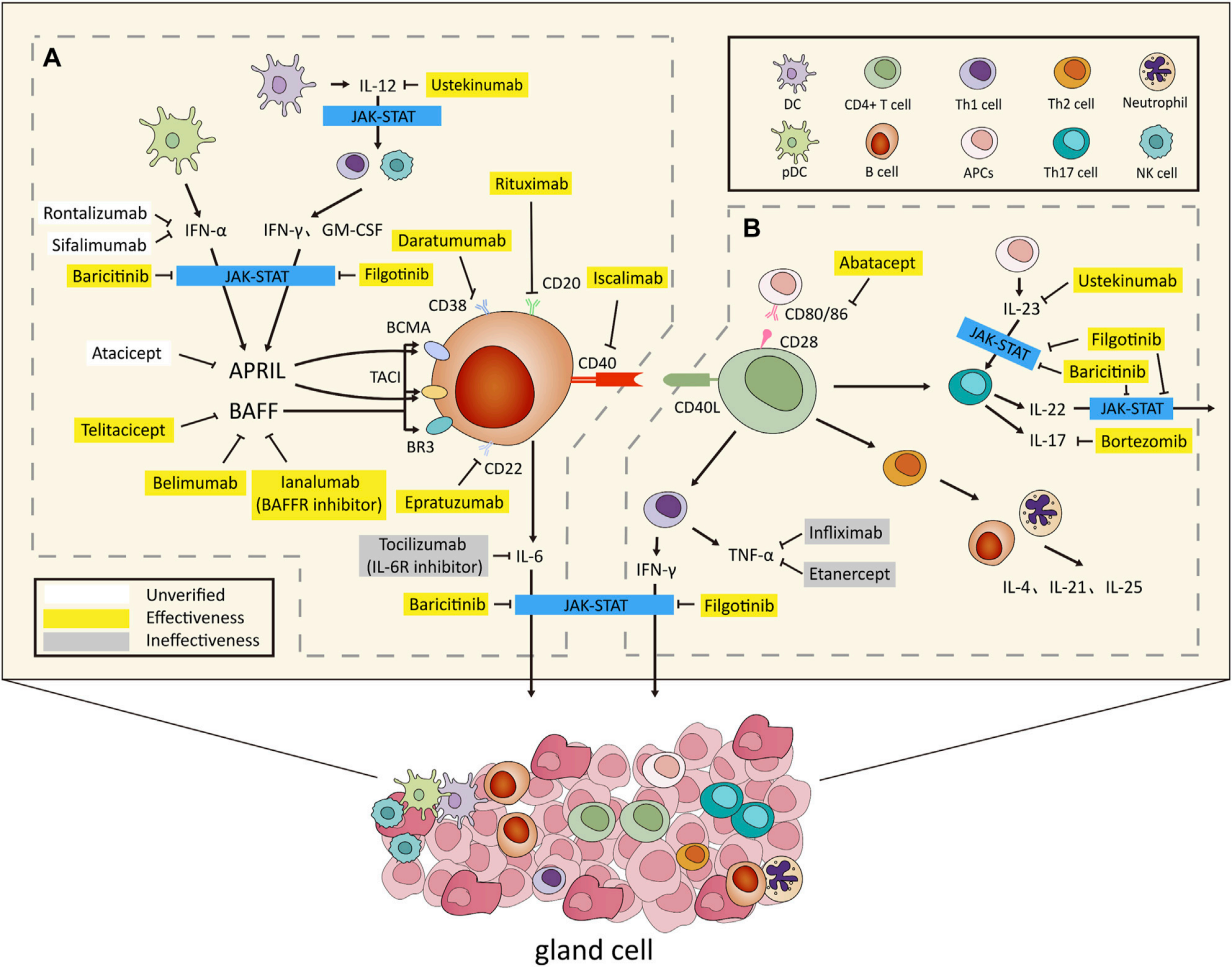
Overview of the Pathogenesis and Biologic Treatments for Sjögren’ s Syndrome. **(A)** After environmental stimulation, pDCs secrete IFN-α, while conventional DCs release IL-12. This triggers IFN-γ and GM-CSF production via innate and adaptive immunity. IFN-α and IFN-γ jointly induce BAFF, promoting B cell activation and IL-6 secretion. APRIL, regulated by IFN-α and IFN-γ, contributes to B cell proliferation. **(B)** CD4+ Th cells, combined with CD80/86, activated by APCs, infiltrate organs, producing cytokines inducing B cell activation. Th1 secretes IFN-γ, Th2 targets B cells and neutrophils, secreting IL-4, IL-21, and IL-25, while Th17, stimulated by IL-23, secretes IL-17 and IL-22. In glands and lymphoid tissues, T and B cells activate with CD40/CD40L. IFN-α, IFN-γ, IL-6, IL-22, and IL-23 use the JAK-STAT pathway for effects. Activation results in gland destruction and extraglandular symptoms, revealing the complex immune interplay causing autoimmune manifestations. Several biologics have been developed to target key factors in this process. Notable examples include rituximab (anti-CD20 monoclonal antibody), epratuzumab (anti-CD22 monoclonal antibody), daratumumab (anti-CD38 monoclonal antibody), belimumab (BAFF inhibitor) and ianalumab (BAFFR inhibitor), telitacicept (BAFF and APRIL inhibitor), iscalimab (anti-CD40 monoclonal antibody), abatacept (anti-CD80/86 monoclonal antibody), infliximab and etanercept (anti-TNF-α monoclonal antibodies), tocilizumab (IL-6R receptor inhibitor), bortezomib (IL-17 receptor inhibitor), ustekinumab (IL-12/23 receptor inhibitor), and baricitinib and filgotinib (JAK inhibitors). These targeted biologics offer specific interventions for autoimmune conditions. Abbreviations: pDCs, plasmacytoid dendritic cells; IFN, interferon; DCs, dendritic cells; GM-CSF, Granulocyte-macrophage colony-stimulating factor; BAFF, B cell-activating factor; IL, interleukin; APRIL, a proliferation-inducing ligand; Th cells, T helper cells; APC, antigen-presenting cell; JAK, Janus kinases; STAT, signal transducer and activator of transcription. In this diagram, white color blocks denote medications not utilized in SS, categorized as unverified. Yellow denotes pharmacological interventions with established efficacy, whereas gray designates medications administered but determined to be ineffective.

Recognized increasingly for diverse systemic complications affecting various organs, cognitive impairments, and persistent fatigue, SS’s pathogenesis supports a molecular-target-based approach to biologic therapy, as illustrated in Figure 1. This approach emphasizes the unique cellular invasion by mononuclear cells, notably CD4^+^ T lymphocytes, into lacrimal and salivary glands, correlating with diverse extraglandular manifestations and underlying autoimmune foundations characterized by elevated cytokine production, aberrant B cell activation, and heightened risk of B cell-derived malignancies (Theander et al., 2011; Nocturne and Mariette, 2018).

In recent years, the treatment landscape for SS has experienced a significant paradigm shift, highlighted by the emergence of innovative biologic drugs. Rigorous clinical exploration, especially targeting molecules critical to SS’s pathogenesis, has proven effective in alleviating both glandular and systemic symptoms. However, the path of these therapeutic approaches through clinical trials and regulatory approval has been complex. Many large randomized controlled trials (RCTs) exclude patients with various extraglandular manifestations, ranging from neurological to pulmonary symptoms, due to the complexity of managing and measuring treatment effects on these diverse and systemic issues (Rihl et al., 2009). This exclusion significantly limits the applicability and relevance of trial results to a broader spectrum of SS patients, often relegating the diverse array of extraglandular symptoms to the margins of research and clinical focus (Ramos-Casals et al., 2020).

Nevertheless, This comprehensive review aims to navigate the evolving landscape of biologic interventions for SS, focusing keenly on the unexplored territories of extraglandular pathology. By shedding light on novel molecular targets and recent breakthroughs in clinical trials, the review seeks to unveil the latent potential of biologic therapies in reshaping the holistic management of both glandular and extraglandular manifestations. Inherent to this discussion is the oft-neglected facet of SS management-the intricate treatment of extraglandular symptoms encompassing fatigue, depression, and anxiety. Scrutinizing the potential of pioneering biologic agents, the review endeavors to illuminate new strategies for effectively addressing these multifaceted challenges.

## 2 Heterogeneity of SS: In-depth analysis from multiple perspectives

Given the complexity and variability of autoimmune diseases, deeper research and classification were needed to improve treatment outcomes and prognosis evaluation. Several large-scale studies have explored the heterogeneity of patients with SS from diverse perspectives, to enhance the precision and personalization of treatment and management for these individuals.

### 2.1 Based on immune dysregulation patterns

PRECISESADS is a project aimed at reclassifying systemic autoimmune diseases based on molecular features determined using various omics platforms (Barturen et al., 2018). In the previous PRECISESADS IMI JU project, it was noted that systemic autoimmune diseases can be categorized into four disease clusters: ‘inflammatory’, ‘lymphoid’, ‘interferon’, and ‘healthy-like’ patterns. Each cluster includes all diagnoses and is defined by genetic, clinical, serological, and cellular features (Barturen et al., 2021). These clusters contribute to the heterogeneity in the initiation, propagation, and flares of the diseases.

Recently, the research team led by Professor Jacque Olivier Pers employed data collected from the PRECISESADS project to investigate immune dysregulation patterns in patients with pSS. This research aims to provide a comprehensive molecular understanding that could inform personalized treatment approaches (Soret et al., 2021). The research involved 300 patients with pSS and matched healthy volunteers. Comprehensive multi-omics analyses were performed on whole blood samples, which included transcriptomics, genomics, epigenetics, cytokine expression, and flow cytometry, incorporating detailed clinical parameters. This study has established a framework to enhance our comprehension of the pathogenic mechanisms in pSS, emphasizing the substantial heterogeneity of the disease.

In their analysis of molecular heterogeneity among pSS patients, researchers identified four distinct patterns of immune dysregulation—C1, C2, C3, and C4—each corresponding to varying disease manifestations and potential treatment approaches. Patients from C2 displayed a healthy-like profile, which has a lower EULAR Sjögren’s Syndrome Disease Activity Index (ESSDAI) compared to the 3 other clusters, but they still experienced the objective symptoms of dryness, pain, and fatigue. Type I interferon (IFN) has traditionally been regarded as the principal factor in the pathogenesis of SS, however, Type II IFN also contributes significantly to the pathogenesis of the disease (Nezos et al., 2015). Notably, C1 and C3 patterns exhibited a significant upregulation of IFN signaling. C1 patients demonstrated the highest scores for both Type I and Type II IFN, while C3 also showed overexpression in the lymphoid pathway, the quantity of peripheral blood B cells was higher compared to other groups. Additionally, C1 and C3 were associated with elevated blood protein levels of C-X-C motif chemokine ligand 10 (CXCL10)/IP-10 (James et al., 2020). Conversely, the C4 pattern prominently expressed signals associated with inflammation and myeloid transcription, it was further characterized by significant lymphopenia and elevated neutrophil levels, with the neutrophil-to-lymphocyte ratio (NLR) previously demonstrated to correlate with disease activity in systemic autoimmunity (Toro-Domínguez et al., 2019; Han et al., 2020). Further analysis uncovered significant differences in serological features and disease activity among these patterns. C1 and C3 showed high levels of hypergammaglobulinemia and anti-ENA antibodies. C4 had intense inflammation and myeloid transcription signals, with the most severe clinical symptoms and disease activity, which have the highest levels of ESSDAI and Patient Global Assessmetn (PGA). These findings provide a theoretical basis for employing Type I IFN inhibitors as therapeutic agents in groups C1, C3, and C4. Specifically for group C3, targeting B cells in treatment strategies appears to yield enhanced therapeutic efficacy. All these findings shed light on the intricate immunological diversity inherent in the SS patients cohort. Crucially, distinct therapeutic strategies emerged for different patterns. The study offers insights into personalized treatment directions, including the inhibition of the IFN signaling pathway, targeted interventions in the lymphoid pathway, and specific interventions in inflammation and myeloid transcription. This theoretical foundation provides a solid basis for the future development of precision medicine and immunotherapy, underscoring the importance of heterogeneity in pSS management.

Currently, the biological diagnosis of SS primarily relies on anti-Ro60/SSA antibodies (Shiboski et al., 2017). The PRECISESADS database was employed for the association between anti-Ro52/TRIM21 antibodies and SS. Participants were classified into four distinct groups based on antibody presence: double-positive (Ro52^+^/Ro60^+^), double-negative (Ro52^−^/Ro60^−^), and two single-positive groups (Ro52^+^)/(Ro60^+^). Patients who were double-positive exhibited more pronounced parotid gland enlargement and elevated β2-microglobulin levels. These patients also had more severe disease activity and higher ESSDAI scores (Bettacchioli et al., 2023). Transcriptomic analyses suggest that the presence of a greater number of identified antibodies correlates with stronger IFN signaling in patients. Therefore, the presence of anti-Ro52/TRIM21 antibodies can be linked to the activation of the interferon pathway. Consequently, these antibodies can serve as valuable markers for assessing the severity and prognosis of SS in affected patients.

### 2.2 Based on clinical phenotypes

In the study conducted by Jessica R. Tarn et al. (Tarn et al., 2019), 608 patients with SS was systematically classified into four subgroups based on the severity of five prevalent symptoms of pSS: pain, fatigue, dryness, anxiety, and depression. These subgroups were designated as low symptom burden (LSB), high symptom burden (HSB), dryness-dominant with fatigue (DDF), and pain-dominant with fatigue (PDF). The DDF subgroup had significant symptoms of dryness and fatigue, as well as decreased anxiety and depression, displaying the most significant glandular dysfunction, with elevated levels of CXCL13, β2-microglobulin, and κ-free light chains (κ-FLC) compared to other subgroups. The PDF subgroup experienced significant symptoms of pain and fatigue, with reduced anxiety and depression levels. Transcriptomic data analysis across the subgroups indicated that both the LSB and DDF subgroups showed increased expression of IFN module activity. And the DDF subgroup also exhibited enhanced activity in the mature B-cell modules, a correlation that likely contributes to the severe glandular dysfunction observed in this subgroup. Furthermore, the DDF subgroup exhibited the highest incidence of lymphoma, highlighting its distinct clinical and pathological profile. These findings were subsequently validated in patient cohorts from France and Norway, confirming the universality and efficacy of the symptom-based patient stratification model.

To ascertain whether the heterogeneity based on clinical symptoms can be effectively applied in the treatment and management of SS, different medications were trialed across various subgroups, reflecting their distinct characteristics. The results demonstrated that hydroxychloroquine was effective in the HSB subgroup, while rituximab showed therapeutic efficacy in the DDF subgroup. The findings demonstrate that classification based on clinical symptom heterogeneity is highly significant for the selection of clinical pharmacotherapy.

### 2.3 Based on histological phenotypes

It is well understood that the profile of infiltrating cells in the salivary glands (SG) of patients with SS evolves as the disease progresses. In the early stages of pSS, when infiltration is mild, the lymphocytic infiltration of the salivary and lacrimal glands is predominantly composed of CD4^+^T cells (Voulgarelis and Tzioufas, 2010). These cells contribute to disease progression by producing pro-inflammatory cytokines such as IFN-γ, IL-17, and IL-21, and by inducing B cell activation. As the disease advances to later stages, with more severe infiltration, B cells begin to dominate the pathological landscape (Verstappen et al., 2021a). This shift, associated with more severe tissue infiltration and exacerbated by the activation and proliferation of B cells, underscores a critical transformation in the immunological landscape of pSS. Furthermore, the degree of lymphocytic infiltration in the salivary glands correlates with the severity of the disease. This transformation highlights the potential for interventions targeting these specific cellular transitions, suggesting that treatments for pSS should consider the dominant immune cells at different disease phases, adopting a stage-dependent approach.

To effectively manage the progression of diseases such as SS, treatment strategies should adapt to the predominant immune cells at each stage. In the early stages of the disease, the therapeutic focus might be on suppressing T-cell activity. Agents that inhibit T cell signaling pathways, such as calcineurin inhibitors such as Cyclosporine or more targeted biologics like anti-CD4 antibodies, can be considered. Since Th1 and Th17 cells are active in this phase, medications that inhibit pro-inflammatory cytokines produced by these cells, such as IFN-γ and IL-17, could also be effective. In the later stages of the disease, when B cells become dominant, the treatment strategy should shift towards interventions targeting B cells. Biologics that target B cells, such as Rituximab, or drugs that inhibit BAFF, such as Belimumab, may be employed. This approach acknowledges the dynamic nature of immune cell involvement in disease progression and tailors treatment to the specific characteristics and demands of each disease stage.

## 3 Current therapies for extra glandular symptoms in SS

The ESSDAI serves as a vital metric in evaluating disease activity across 12 domains (including Lymphadenopathy and lymphoma, articular, cutaneous, pulmonary, renal, muscular, central and peripheral neurological, and hematological domains) (Ramos-Casals et al., 2020). In a study of 921 patients with SS in Spain, only 8% of had no disease activity according to ESSDAI scoring. The highest cumulative scores were in the articular, pulmonary, haematological, and peripheral nervous system domains (Ramos-Casals et al., 2014).

This indicates that extraglandular symptoms are prevalent among SS patients, and a significant majority concurrently endure both glandular and extraglandular manifestations. However, the mechanisms behind the glandular and extraglandular effects of SS are not yet clear. Therefore, treatments for SS often focus on easing the symptoms rather than directly addressing the root cause of the disease.

Salivary gland epithelial cells (SGECs) have recently been recognized for their critical role in SS. Notably, SS is characterized by a significant reduction in salivary gland function, a decline partly attributable to specific deficits in the glandular epithelium (Verstappen et al., 2021b). Additionally, SGECs may also play a significant role in extraglandular symptoms.

A study revealed that SGECs from patients with primary SS (pSS) possess a unique capacity to enhance the survival and activation of B-lymphocytes (Rivière et al., 2020). Epithelial cells secrete pro-inflammatory cytokines and autoantigens, leading to the infiltration and activation of T and B cells, which significantly contribute to inflammation and tissue damage (Verstappen et al., 2021b). Recent research has shown that cytokines secreted by dysfunctional glandular epithelial cells, such as TNF-α and IFN-γ, can disrupt the junctional structure of the epithelium. This disruption facilitates the circulation of these cytokines and triggers inflammatory responses in peripheral tissues, thus providing a potential explanation for the connection between glandular dysfunction and systemic symptoms, including joint pain and fatigue (Negrini et al., 2022). Additionally, according to Katsiougiannis et al., epithelial cells in SS exhibit abnormal expression of autoantigens and adhesion molecules, potentially provoking an autoimmune response not just locally but systemically (Katsiougiannis et al., 2019).

### 3.1 Musculoskeletal involvement

SS is associated with both joint and muscle manifestations, including arthralgia and arthritis, as well as myopathy, which is often asymptomatic. As for joints, approximately 50 percent of patients with primary SS report arthralgia, with or without evidence of arthritis (Pease et al., 1993). Current research indicates that patients positive for rheumatoid factor (RF) or anti-citrullinated peptide antibodies (ACPA) generally suffer from a more severe form of inflammatory arthritis, which is often erosive and poses an increased risk of evolving into rheumatoid arthritis (RA) (Kamali et al., 2005; Mohammed et al., 2009; Ryu et al., 2013; Molano-González et al., 2019). Additionally, in a comprehensive study, around 40 percent of SS patients tested positive for RF, and ACPA are found in 5–10 percent of patients with pSS (Payet et al., 2015).

Concerning muscle involvement, SS can lead to mild inflammatory myopathy, presenting either as subclinical symptoms or gradual proximal muscle weakness. More than 70 percent of SS patients experience myalgias. However, a detailed study of 395 SS patients in France revealed that of 38 initially suspected of having myositis, only four cases were confirmed (Felten et al., 2021a). Additionally, antibodies to cytosolic 5′-nucleotidase 1A, a marker for inclusion body myositis, are detectable in nearly half of the SS patients without manifest muscle disease (Rietveld et al., 2018).

The management of musculoskeletal symptoms in SS primarily aims at providing symptomatic relief. For mild joint symptoms or when patients exhibit only arthralgia and myalgia without inflammatory synovitis, non-steroidal anti-inflammatory drugs are typically prescribed. In cases of moderate to severe arthritis, methotrexate (MTX) and hydroxychloroquine (HCQ) are the treatments of choice (Price et al., 2017). Biologic agents such as rituximab (RTX) or tumor necrosis factor-α (TNF-α) inhibitors are reserved for a small subset of refractory cases, particularly in patients with overlapping features of RA (Carsons et al., 2017).

### 3.2 Lung involvement

In 9%–20% of cases, SS is associated with various respiratory symptoms. The pulmonary lesions in these instances are typically determined by symptoms, pulmonary function tests, or abnormalities observed in chest radiographs. The most common manifestations are chronic interstitial lung disease (ILD) and tracheobronchial disease (Flament et al., 2016). Among them, non-specific interstitial pneumonitis (NSIP) emerges as the predominant histopathologic anomaly. However, similar lesions may also appear in other immune disorders, necessitating an investigation into the possibility of coexisting rheumatic diseases. Notably, patients with ILP often exhibit either polyclonal or monoclonal gammopathy (American Thoracic Society, 2000).

The management of ILD in challenging due to the lack of histological correlation with known factors. To date, therapeutic options for SS patients are mostly empirical, and there is a significant need for evidence-based recommendations for treating pulmonary manifestations. Currently, oral prednisone is the treatment of choice for ILD. If patients exhibit intolerance or non-responsiveness to glucocorticoids, alternatives like azathioprine and mycophenolate mofetil (MMF) may be beneficial. Additionally, biologics such as nintedanib, proven effective in managing ILDs associated with connective tissue disorders, including SS. Some studies also indicate potential improvement in pulmonary symptoms with RTX. However, its efficacy in SS-related pulmonary conditions remains limited (Luppi et al., 2020). Moreover, shared transcriptional activities in fibroblasts between SS and interstitial pneumonia suggest fibroblasts as potential therapeutic targets (Jin et al., 2019; Korsunsky et al., 2022).

### 3.3 Skin involvement

Cutaneous involvement is a relatively common manifestation of SS, presenting with various symptoms such as xeroderma, eyelid dermatitis, annular erythema, and cutaneous vasculitis (Generali et al., 2017). Xeroderma, or skin dryness, is the most common skin manifestation of SS, afflicting 67% of patients (Roguedas et al., 2004). The pathogenesis of xerosis may involve alterations in the stratum corneum, the outermost layer of the skin, coupled with decreased secretion from sebaceous and sweat glands (Bernacchi et al., 2004; Bernacchi et al., 2005). Unlike atopic dermatitis, SS-related dry skin exhibits unique pathophysiological features, including changes in keratins and total skin proteins (Bernacchi et al., 2005; Katayama, 2016).

Recent studies have supported the role of T helper 17(Th17) cells in SS-related skin conditions, evidenced by elevated levels of Interleukin-17 (IL-17), IL-6, and IL-12 (Grisius et al., 1997; Manoussakis et al., 2007; Nguyen et al., 2008; Yoshimoto et al., 2011; Ciccia et al., 2012; Ciccia et al., 2015; Fogel et al., 2018). The majority of these factors are related to T-cells. Therefore, for patients with relevant skin lesions, it is worth considering whether T-cell targeted therapies and inhibitors of related factors should be the preferred treatment options. Currently, the most effective approach for mitigating pruritus in SS including skin moisturization, reducing the frequency of bathing, application of body lotion, and avoiding medications with anticholinergic effects. When these measures are insufficient, antihistamines such as cetirizine, fexofenadine, and famotidine should be considered. In instances resistant to conventional treatments, oral corticosteroids may be used. If these interventions fail to alleviate pruritus, screening for primary biliary cholangitis (PBC) is crucial, which is a frequent extrahepatic manifestation of SS (Chalifoux et al., 2017).

### 3.4 Liver involvement

SS can be associated with liver abnormalities, which typically include mild biochemical indicator deviations and histological changes indicative of PBC or autoimmune hepatitis. Patients diagnosed with PBC exhibit a prevalence of pSS of approximately 38%. However, clinical evidence of PBC is observed in fewer than 2% of patients with SS, according to large cohort studies (Trevisani et al., 2022). In instances where SS is accompanied by liver dysfunction, treatment usually involves the isolated use of hepatoprotective drugs such as ursodeoxycholic acid, aimed at symptomatically improving liver function. This approach does not emphasize the routine use of conventional medications for treating SS.

### 3.5 Psychological involvement

A gap exists in the literature concerning a comprehensive theoretical model that explains the emergence of psychological symptoms such as fatigue, depression, and anxiety in patients with SS. Nonetheless, existing research has highlighted correlations with certain factors. Fatigue in SS correlates with factors like IL-1, IL-36α, and humoral autoimmunity-related components (Zeng et al., 2022), while inversely correlating with pro-inflammatory cytokines, including IFN-γ, TNF-α, lymphotoxin α, and CXCL10 (Davies et al., 2019). Research also suggests involvement of the hypothalamic-pituitary-adrenal (HPA) axis in fatigue development, potentially due to autoimmune-mediated adrenal gland destruction by autoantibodies (Mæland et al., 2021). An RCT conducted in Brazil provided support for this association, demonstrating that enhancement of cortical excitability via transcranial direct-current stimulation could reduce fatigue in SS patients (Pinto et al., 2021).

Currently medications such as HCQ, dehydroepiandrosterone, and RTX have proven ineffectiveness in alleviating fatigue in controlled studies (Hartkamp et al., 2008; Devauchelle-Pensec et al., 2014; Mariette et al., 2015a). Ongoing clinical trials explore novel fatigue treatment targets in primary SS, including experimental drugs like Lanraplenib (spleen tyrosine kinase inhibitor), Filgoinib (Janus kinase-1 inhibitor), Tirabrutinib (Bruton’s tyrosine kinase inhibitor), CDZ173 (PI3K inhibitor), and Dazodalibep (CD40L antagonist and Tn3 fusion protein) (Mæland et al., 2021). However, Lanraplenib, Filgotinib, and Tirabrutinib have not shown significant benefits over placebo (Price et al., 2022), whereas Dazodalibep showed promising effects in reducing fatigue (St et al., 2023). RSLV132, an RNaseFc fusion protein, is also showing promise in fatigue management in a phase II investigation (Posada et al., 2021).

Depression in SS is potentially linked to disruptions in neurotransmitter functionality and brain-derived neurotrophic factor, with additional considerations given to the intestinal microbiota, amino acid metabolism, and neuropeptide-targeting autoantibodies (Caspani et al., 2019; Tian et al., 2020). Anxiety correlates with autoantibodies against α-melanocyte-stimulating hormone (Karaiskos et al., 2010), and may be exacerbated by B-cell activating factor (BAFF), which contributes to brain inflammation, neuronal impairment, and hippocampal remodeling (Crupi et al., 2010). N-3 polyunsaturated fatty acids have shown potential in inhibiting neuroinflammation associated with depressive states in SS (Crupi et al., 2012).

Research continues into anti-BAFF drugs for anxiety and selective serotonin reuptake inhibitors for depression, given their potential roles in the inflammatory processes associated with SS-related fatigue (Schlesinger et al., 2011; Vivino et al., 2016; Bodewes et al., 2019). Additionally, contemporary research is exploring the impact of Chinese medicine on anxiety, depression, and overall quality of life in patients with pSS (Wu and Li, 2020).

### 3.6 Hypergammaglobulinemia and hypogammaglobulinemia

Hypergammaglobulinemia is common in patients with SS and is presented in almost half of the patients (Ramos-Casals et al., 2002; Yang et al., 2018). It can manifest as either polyclonal or monoclonal. The presence of hypergammaglobulinemia is closely associated with the presence of anti-Ro/SSA and anti-La/SSB antibodies and RF (Alexander et al., 1983; Ramos-Casals et al., 2002). Hypogammaglobulinemia is less prevalent than hypergammaglobulinemia, and may also develop in patients with established SS as a sign of underlying lymphoma (Anderson and Talal, 1972).

Patients with SS can also experience Monoclonal gammopathies (MG). Monoclonal IgG proteins are the most frequently detected class, followed by IgM. When SS is complicated by MG, patients often exhibit a higher incidence of abnormal urine NAG, higher levels of ESR, ESSDAI, and Clinical ESSDAI (ClinESSDAI) scores (Yang et al., 2018). Multivariate analysis revealed that the disease activity, assessed by either ESSDAI or ClinESSDAI, was the sole independent risk factor for the presence of MG (Yang et al., 2018).

### 3.7 Exploring alternative approaches

Recent clinical trials have explored novel treatments in pSS, encompassing experimental drugs like Lanraplenib (spleen tyrosine kinase inhibitor), Filgoinib (Janus kinase-1 inhibitor), Tirabrutinib (Bruton’s tyrosine kinase inhibitor), CDZ173 (PI3K inhibitor), and Dazodalibep (CD40L antagonist and Tn3 fusion protein) (Mæland et al., 2021). However, neither Lanraplenib, Filgotinib, nor Tirabrutinib demonstrated significant differences from placebo in improve the clinical SS-related symptoms, ESSPRI and ESSDAI scores (Price et al., 2022). Conversely, Dazodalibep showed a significant reduction in disease activity measured by the improvement in ESSDAI score (St et al., 2023). Contemporary research aims to assess the impact of Chinese medicine on anxiety, depression, and overall quality of life in pSS patients (Wu and Li, 2020). Current pharmaceutical strategies, including hydroxychloroquine, RTX, and TNF-α inhibitors, consistently show significant improvements in extraglandular symptoms like fatigue. Ongoing explorations of anti-BAFF drugs for anxiety and selective serotonin reuptake inhibitors for depression are underway, considering factors like IL-1, IL-36α, and humoral autoimmunity in SS-related fatigue (Schlesinger et al., 2011; Vivino et al., 2016; Bodewes et al., 2019), with recent studies revealing an inverse correlation between fatigue intensity and pro-inflammatory cytokines, including IFN-γ, TNF-α, lymphotoxin α, and CXCL10 (Davies et al., 2019).

In SS, the functionality of the salivary glands is frequently substantially diminished. Consequently, in the treatment of patients with this dry syndrome, the restoration of salivary gland function should be a top priority. However, current therapeutic approaches primarily target glandular inflammation and may be insufficient to address the restoration of salivary gland function (Gueiros et al., 2019). In the existing studies, the inhibition with anti-BAFF, anti-APRIL, anti-IL-6R antibodies, Janus kinases1/3 (JAK1/3) inhibitor, or hydroxychloroquine did not exhibit any inhibitory effect on active B lymphocytes in SGECs. In contrast, leflunomide, BTK, or PI3K inhibitors all demonstrated favorable effects (Rivière et al., 2020).

## 4 Biologic therapies in SS

### 4.1 B Cell targeting approach

#### 4.1.1 Mechanism

The escalating interest in biologic therapies is grounded in the recognition of B cell hyperactivity as a central facet of SS pathogenesis. SS is distinguished by heightened B cell activity contributing to autoimmune-mediated glandular impairment, with approximately 35%–40% of patients manifesting hypergammaglobulinemia, thereby presenting symptoms of xerophthalmia and xerostomia (Nocturne and Mariette, 2018; Du et al., 2021). B cells are crucial for producing autoantibodies associated with SS, including ANA, anti-SSA, and anti-SSB antibodies, which are central to the autoimmune responses observed in the syndrome. In pSS, the activation of B cells can lead to the overproduction of κ and λ light chains, which are then secreted into the serum as free light chains (FLC). Serum FLC levels in pSS are associated with IgG, RF, and systemic disease activity, indicating their potential as biomarkers for monitoring the disease. Additionally, chronic activation of B cells may heighten the risk of developing lymphomas, particularly mucosa-associated lymphoid tissue-lymphoma (MALT-L) (Du et al., 2021).

CD20, expressed on B cell precursors, is pivotal in B cell activation, proliferation, and differentiation, making it a rational target for addressing the B cell dysfunction underpinning SS. In this context, RTX, a chimeric monoclonal antibody directed against CD20, emerged as a prospective therapeutic option (Gurcan et al., 2009). Furthermore, B cells are responsible for the secretion of a myriad of cytokines that contribute to the pathogenesis and advancement of SS, including but not limited to IL-4, IL-6, IL-10, and various others (Ohyama et al., 1996; van Woerkom et al., 2005).

#### 4.1.2 Application of rituximab in SS

Rituximab (RTX), a chimeric monoclonal antibody directed against CD20, that achieves therapeutic effects by reducing the number of circulating B cells (Beers et al., 2010) and modulating T cell responses in autoimmune diseases (Ciccia et al., 2013; Ciccia et al., 2014), has emerged as a frontrunner in alleviating symptoms such as fatigue and oral dryness. The application of RTX in SS aims to modulate aberrant B cell responses (Meijer et al., 2010). The combination of RTX with bendamustine has been evaluated in MALT-L complicating pSS. Several studies have demonstrated its efficacy and safety in low-grade B-cell lymphomas, including mantle cell lymphomas and extra gastric MALT-L (Rummel et al., 2013; Salar et al., 2014). These investigations have yielded a spectrum of outcomes, with variable improvements observed in measures such as fatigue, salivary flow rates, and joint pain (Dass et al., 2008; Meiners et al., 2012; Gottenberg et al., 2013). However, the outcomes have not been consistently uniform, and several trials have failed to meet their primary endpoints (Chen et al., 2021), with concerns about safety and long-term efficacy (Table 1). According to current RCTs, RTX demonstrated no significant differences compared to the placebo in terms of pain, fatigue, and dry mouth (Devauchelle-Pensec et al., 2014; Bowman et al., 2017). As a result, we do not recommend the use of RTX for the treatment of ocular dryness, pain, or fatigue (Ramos-Casals et al., 2020). Recent studies indicate that patients with elevated levels of B-cell infiltration in the parotid glands tend to exhibit a more favorable response to RTX. This implies that, in the future, we may be able to tailor drug treatments more precisely for individual patients (Delli et al., 2016).

**TABLE 1:** Summary of Sjögren’s Syndrome therapeutic targets and biologics treatments.

Target	Duration	Evaluation criterion	Study type	Treatment	No. of subjects	Finding	Refs
**B cell**							
CD20	48 weeks	The count of IL-22+ cells in pSS patients’ SG.	L	RTX	10 pSS	RTX in pSS reduces salivary gland IL-22, potentially affecting lymphoma progression	Bernacchi et al. (2004), Ciccia et al. (2013)
	52 weeks	SG expression of IL-17, IL-23p19, and p-STAT3	L	RTX	15 pSS	RTX globally reduces IL-17 and specifically depletes mast cells in pSS.	Bernacchi et al. (2005), Ciccia et al. (2014)
	48 weeks	Primary: Stimulated saliva flow	RCT	RTX vs. placebo	30 pSS	RTX effectively and safely improves the primary and secondary outcomes	Meijer et al. (2010), Katayama (2016)
		Secondary: B cell and RF levels, MFI scores, VAS scores for sicca symptoms, and extraglandular issues					
	26 weeks	Primary: Fatigue reduction>20%	RCT	RTX vs. placebo	17 pSS	RTX improved fatigue and showed potential benefits in social functioning and mental health in pSS.	Grisius et al. (1997), Dass et al. (2008)
		Secondary: Changes in SF-36 social functioning and trend for SF-36 mental health scores					
CD20	35 months	ESSDAI, corticosteroid reduction, and adverse reactions	O	RTX vs. RTX + ISx	78 pSS	RTX reduces disease activity and corticosteroid dosage in systemic pSS treatment	Manoussakis et al. (2007), Gottenberg et al. (2013)
	60 weeks	ESSPRI and ESSDAI.	O	RTX	28 pSS	ESSPRI and ESSDAI detect pSS treatment changes, with ESSDAI more responsive in rituximab-treated patients	Meiners et al. (2012), Fogel et al. (2018)
CD22	52 weeks	Primary: Efficacy and safety of epratuzumab	RCT (p)	Epratuzumab vs. placebo	113 sSS-SLE	Epratuzumab enhanced disease activity and hastened B cell/IgM reduction in sSS-SLE.	Chalifoux et al. (2017), Gottenberg et al. (2018)
		Secondary: B cell count and IgM level					
CD38	6 months	ESSDAI and ESSPRI.	L	Daratumumab	2 refractory pSS	ESSDAI decreased in both pSS patients, and ESSPRI remained stable	Trevisani et al. (2022), Nocturne et al. (2023)
BAFF	28 weeks	Primary: reduction in dryness, fatigue, pain scores, b cell activation biomarker, and systemic activity	RCT	BEL	30 pSS	60% met the primary endpoint, showing notable reductions in the ESSDAI and improvements in dryness, while salivary flow and Schirmer’s test remained unchanged	Mariette et al. (2015b), Mæland et al. (2021)
		Secondary: ESSDAI and ESSPRI.					
BAFF	68 weeks	ESSDAI and B cell depletion	C&L	BEL + RTX vs. BEL vs. RTX vs. placebo	86 pSS	Combined BEL + RTX induced enhanced salivary gland B cell depletion	Manoussakis et al. (1999), Mariette et al. (2022)
	24 weeks	SG quality assessed by ultrasound	RCT	Ianalumab	27 pSS	Ianalumab treatment improved gland quality, reduced inflammation, and altered perfusion and stiffness *versus* placebo	Diekhoff et al. (2020), Posada et al. (2021)
BAFF/APRIL	24 weeks	ESSDAI, MFI-20, and serum immunoglobulin level	RCT	Telitacicept vs. placebo	42 pSS	The Telitacicept group reduced MFI-20 and immunoglobulin levels, but there was no change in the ESSDAI score	Mariette et al. (2015a), Xu et al. (2023)
**T-B cell interaction**							
CD40	12 weeks	ESSDAI, adverse events	RCT	Iscalimab vs. placebo	44 pSS	Only intravenous iscalimab resulted in a significant reduction in ESSDAI.	Crupi et al. (2012), Clarke (2020)
	20 weeks	Primary: Efficacy and safety of iscalimab	RCT	Iscalimab vs. placebo	82 pSS	Subcutaneous iscalimab did not significantly differ from placebo, yet intravenous treatment notably reduced the ESSDAI score	Schlesinger et al. (2011), Fisher et al. (2020)
		Secondary: ESSDAI.					
**T cell**							
CD80/86	48 weeks	Primary: ESSDAI, ESSPRI.	L	Abatacept	15 pSS	Abatacept treatment decreased the ESSDAI, ESSPRI, RF, and IgG levels, and improved fatigue, health-related quality of life, and disease activity. But the gland function did not change	van Woerkom et al. (2005), Meiners et al. (2014)
		Secondary: RF and IgG levels, fatigue, gland function, adverse events					
CD80/86	52 weeks	Primary: the remission rate as measured by SDAI.	O	Abatacept	68 sSS-RA	Abatacept increased the SDAI remission, ameliorated glandular, extraglandular involvements, systemic disease activities, and patient-reported outcomes	Beers et al. (2010), Tsuboi et al. (2023)
		Secondary: Saxon’s test, Schirmer’s test, ESSDAI, ESSPRI, adverse events					
	169days	Primary: ESSDAI.	RCT	Abatacept vs. placebo	187 pSS	Abatacept decreased the disease-relevant biomarkers (including IgG, IgA, IgM- RF), but there was no change in the clinical efficacy	Ciccia et al. (2013), Baer et al. (2021)
		Secondary: ESSPRI, SWSF, b cell activation biomarker, immune cell phenotypes					
**TNF**							
TNFα	22 weeks	Primary: improvement of disease activity	RCT	Infliximab vs. placebo	103 pSS	Infliximab did not significantly differ from placebo in both primary and secondary endpoints	Mariette et al. (2004), Gottenberg et al. (2013)
		Secondary: the level of CRP, ESR, the number of tender and swollen joints, gland function, and life quality					
TNFα	12 weeks	Primary: Clinical assessments of disease activity	RCT	Etanercept vs. placebo	28 SS	Etanercept did not significantly differ from placebo in the primary clinical outcomes, and can’t decrease the markers of immune activation, frequency of cell subpopulations, and aberrant cytokine profile levels	Moutsopoulos et al. (2008), Meiners et al. (2012)
		Secondary: Peripheral blood distribution of T cells, B cells, monocytes, expression of their activation markers, systemic cytokine levels					
**ILs**							
IL-6	44 weeks	ESSDAI, VNS.	RCT	Tocilizumab vs. placebo	110 pSS	Tocilizumab did not improve systemic involvement and symptoms compared with placebo	Vincent et al. (2014), Felten et al. (2021b)
IL-17	3 months	ESSDAI, fatigue, headache, the levels of ESR, globulins, and serum viscosity	L	Bortezomib	1 refractory pSS	Bortezomib can improve the general symptoms, particularly fatigue, also decrease serum globulin levels and serum viscosity	Brignole et al. (2000), Jakez-Ocampo et al. (2015)
IL-12/23	3 years	The levels of autoimmune antibody, ESR, CRP, gland function, and extraglandular issues	L	Ustekinumab	1 refractory sSS- psoriatic	Ustekinumab can improve psoriasis and joint pain in SS patients with psoriatic	Chimenti et al. (2015), Clarke (2020)
**JAK**							
JAK1/2	6 months	ESSDAI, ESSPRI, PGA scores, IgG and ESR level, remission of organ manifestations	L	Baricitinib	11 pSS	Baricitinib decreased the ESSDAI, ESSPRI, and PGA, improved the symptoms of arthritis, skin rash, and ILD in patients with SS.	Giltiay et al. (2017), Bai et al. (2022)
JAK1	52 weeks	Primary: week-12 proportion of patients fulfilling protocol-specified improvement criteria (based on CRP, SS-related symptoms)	RCT	Filgotinib vs. lanraplenib vs. tirabrutinib vs. placebo	150 SS	Filgotinib reduced Type I IFN signature activity. There were no changes in primary and secondary endpoints	Felten et al. (2021a), Price et al. (2022)
		Secondary: ESSDAI, ESSPRI, adverse events					

L, longitudinal study; RCT, randomized, placebo-controlled trial; O, prospective observational study; C, cross-sectional study; RCT (p), *post hoc* analysis of rendomized, placebo-controlled trial; Primary, primary endpoint; Secondary, secondary endpoint; BAFF, B-cell activating factor; APRIL, a proliferation-inducing ligand; TNF, tumour necrosis factor; IFN, interferon; STAT, signal transducer and activator of transcription; IL, interleukin; JAK, janus kinases; SS, Sjögren’s syndrome; pSS, primary Sjögren’s syndrome; sSS, secondary Sjögren’s syndrome; SLE, systemic lupus erythematosus; RA, rheumatoid arthritis; ILD, interstitial lung disease; sSS-SLE, SLE, with associated SS; sSS-RA, SS, associated with RA; sSS- psoriatic, SS, associated with psoriatic; RTX, rituximab; ISx, immunosuppressant; BEL, belimumab; SG, salivary gland; MFI, multidimensional fatigue inventory; VAS, visual analog scale; SF-36, 36-item Short-Form Health Survey; ESSPRI, the EULAR, Sjögren’s Syndrome Patient Reported Index; ESSDAI, the EULAR, Sjögren’s Syndrome Disease Activity Index; SWSF, stimulated whole salivary flow; SDAI; the Simplified Disease Activity Index; VNS, visual numeric scale; PGA, physician global assessment; RF, rheumatoid factor; CRP, C-reactive protein; ESR, erythrocyte sedimentation rate; wks, weeks; mos, months; vs., verse.

#### 4.1.3 Limitations

The effectiveness of RTX in SS has faced limitations due to the heterogeneous nature of the disease and varying responses among patients. Some autoimmune patients have developed autoimmunity against RTX itself. The intricate interplay of factors influencing SS pathogenesis adds complexity to assessing RTX’s effectiveness in diverse patient profiles. Additionally, understanding the long-term impact of RTX on disease progression and the underlying immune dysregulation requires further investigation. Some studies suggest that the use of RTX is associated with an increased likelihood of adverse events, primarily respiratory infections (Fox et al., 2021).

#### 4.1.4 Exploring alternative approaches

B-cell activating factor (BAFF) and a proliferation-inducing ligand (APRIL) significantly influence B cell maturation, proliferation, and survival (Chen et al., 2021). Multiple studies consistently highlight BAFF’s substantial involvement in SS pathogenesis, emphasizing its critical role (Lavie et al., 2008; Yoshimoto et al., 2011; Loureiro-Amigo et al., 2021). Therapeutic interventions aimed at inhibiting B-cell activation through BAFF disruption have been the subject of extensive research. Notable agents in this context include belimumab, an antibody that targets BAFF (Mariette et al., 2015b), and atacicept or telitacicept, a soluble wild-type extracellular domain Fc fusion protein that strongly inhibits BAFF and weakly inhibits APRIL signaling (Samy et al., 2017; Shi et al., 2021; Xu et al., 2023). It is important to note that the sustained use of BAFF antagonists effectively reduces disease activity but may not impact certain parameters such as salivary flow rate, Schirmer’s test, and lesion scores in salivary gland biopsies (Mariette et al., 2015b).

The BAFF/APRIL system comprises not only these two ligands but also three receptors: BAFF receptor (BAFF-R), also known as B-lymphocyte stimulator receptor 3 (BR3), B-cell maturation antigen (BCMA), and transmembrane activator and cyclophilin ligand interactor (TACI) (Vincent et al., 2014). Both BAFF and APRIL can interact with BCMA and TACI, while BAFF exclusively binds to BAFF-R (Vincent et al., 2012). These receptors also play a pivotal role in the survival, maturation, and regulation of B cells (Vincent et al., 2013). Presently, there is ongoing research on drugs targeting these receptors, Ianalumab, a novel BAFF-R targeting antibody, demonstrated favorable therapeutic effects in primary SS patients, affecting ESSDAI score, gland quality, inflammation, perfusion, and stiffness (Diekhoff et al., 2020; Bowman et al., 2022).

In the pursuit of diversified therapeutic strategies, novel avenues have been explored (Table 1). A notable focus lies in targeting CD22, a protein on B cell surfaces, exemplified by epratuzumab, a humanized IgG1 anti-CD22 antibody. In contrast to the depletion approach of RTX, epratuzumab modulates B cell activity, potentially offering an alternative means to mitigate B cell-mediated autoimmune responses (Giltiay et al., 2017). Epratuzumab enhanced disease activity and hastened B cell/IgM reduction in systemic lupus erythematosus (SLE) patients with SS (Gottenberg et al., 2018). However, further research is required to establish its effectiveness. CD38, a glycoprotein found on plasma cells, acts as an adhesion molecule, ectoenzyme, and receptor for activation or proliferation signals. CD38 antibodies can directly interfere with plasma cells through calcium disruption and signal transduction, or Fc-dependent immune-effector mechanisms (complement-dependent cytotoxicity, antibody-dependent cellular cytotoxicity, and antibody-dependent cellular phagocytosis). Daratumumab, a monoclonal antibody targeting CD38 on multiple myeloma cells and immune cells, is being explored for potential applications in autoimmune diseases such as SS (Nocturne et al., 2023). Its mechanism of action in modulating the autoimmune response shows promise, but rigorous clinical trials are essential to establish its safety and efficacy in these specific conditions.

Iguratimod, by reducing Immunoglobulins production and suppressing B cell proliferation, proved effective and well-tolerated in a 24-week clinical trial with 66 pSS patients. It ameliorated dryness symptoms and disease activity, concurrently decreasing BAFF levels and plasma cell proportions (Li et al., 2019). CD40 has been identified as involved in a spectrum of cell-mediated responses and has been implicated in the pathogenesis of chronic inflammatory disorders (Dimitriou et al., 2002) Studies have shown elevated CD40 expression in salivary gland and conjunctival cells of SS patients, indicating its involvement in the disease. CD40 has also been found to be upregulated in the conjunctival cells of SS patients with dry eyes (Brignole et al., 2000). Recently, Iscalimab, an anti-CD40 monoclonal antibody, exhibited preliminary efficacy in treating pSS in a phase II clinical trial (Clarke, 2020; Fisher et al., 2020). The intricate nature of SS underscores the need for ongoing research into these therapeutic modalities to optimize their clinical application and address the complexities of autoimmune pathogenesis.

Remibrutinib is a selective, covalent inhibitor of Bruton’s tyrosine kinase (BTK). By inhibiting BTK activity, it reduces B-cell-mediated inflammation and autoimmune responses. A recent Phase II RCT demonstrated that remibrutinib significantly improved the ESSDAI scores compared to placebo. Additionally, it enhanced salivary flow rates. These findings substantiate the potential of remibrutinib as a therapeutic option for SS and provide a rationale for further drug development and extended-duration trials (Dörner et al., 2024).

#### 4.1.5 Exploring B Cell targeted approaches and potential synergies

Biologic therapies targeting B cell dysregulation continue to evolve in the management of SS. While RTX’s clinical outcomes show variability, ongoing research endeavors aim to optimize its application. Simultaneously, alternative strategies like CD22 targeting and BAFF interference hold promise in reshaping the treatment landscape. Currently, two considerations underlie the diverse clinical outcomes associated with the use of RTX: the reconstitution of B cells and the increased levels of BAFF after B-cell depletion.

The restoration of peripheral blood B cells following B-cell depletion with RTX mirrors the developmental process of B cells and typically begins between 6 and 12 months after the last infusion. B-cell reconstitution has been widely described in numerous autoimmune diseases, such as Sjögren’s Syndrome (SS), systemic lupus erythematosus (SLE), rheumatoid arthritis (RA), immune thrombocytopenia (ITP) (Abdulahad et al., 2011; Crickx et al., 2020). The second hypothesis posits that, upon depletion of B cells, the diminished B cell count leads to increased unbound BAFF levels, fostering a microenvironment conducive to B cell regeneration (Zhang et al., 2023). This hypothesis is supported by an experimental setting, serum analysis of five patients, including two with SS, who underwent RTX treatment, revealed an early elevation in BAFF levels (Lavie et al., 2007).

Given the current circumstances, it is worthwhile to further explore the potential of combining anti-B cell and anti-BAFF therapies for synergistic treatment of SS. Encouragingly, clinical trials investigating the combination of RTX and belimumab for the treatment of pSS are currently underway, providing fertile ground for further therapeutic advancements in the field (De Vita et al., 2014; Gandolfo and De Vita, 2019). Presently, data from a phase II clinical trial demonstrate that the combination of belimumab and RTX induces a more pronounced depletion of B cells in the salivary glands compared to monotherapies, potentially resulting in improved clinical outcomes (Mariette et al., 2022). Furthermore, in this experimental setting, the sequential treatment protocol begins with the administration of Belimumab, followed by the subsequent addition of RTX. This strategy aims to preemptively disrupt the environment conducive to B cell growth before commencing the depletion of B cells, with the potential of achieving enhanced therapeutic efficacy.

### 4.2 T cell-targeted therapies in SS

#### 4.2.1 Mechanism

B cells have long been considered critical in the pathogenesis of SS, yet studies indicate that their hyperactivity is influenced by T cells (Verstappen et al., 2021a). Furthermore, T lymphocytes and the cytokines they release, including IL-17, IL-22, and various other factors, occupy a pivotal role in the initiation and perpetuation of inflammatory infiltrates within the salivary glands of individuals affected by SS (Katsifis et al., 2007; Ciccia et al., 2012). Particularly in the early stages of the disease, CD4^+^ T cells are the primary lymphocytes infiltrating the salivary and lacrimal glands. Consequently, investigating the pathological mechanisms of T cells in SS is as vital as studying the B cells.

Current research indicates that various Th cell types formed after the differentiation of CD4^+^ T cells produce different signaling factors. Notably, studies have detected aberrant expression of the IL-22 receptor in mantle cell lymphoma (MCL), which may promote cell growth in MCL by modulating various cellular signaling pathwaysThis observation leads to the hypothesis that patients with increased IL-22/IL-22R pathway expression might be at a higher risk for lymphoma (Gelebart et al., 2011). T-cell immune responses involve the formation of specialized junctions between T cells and antigen-presenting cells mediated by adhesion molecules. In SS, autoantigens on endothelial cells trigger T-cell migration to exocrine tissue, leading to activation and increased B-cell autoantibody production (Zhou et al., 2020). This mechanism has spurred interest in developing drugs that target adhesion molecules to modulate T-cell responses in SS.

#### 4.2.2 Application of abatacept in SS

Current research suggests that CD4^+^ T cells are the primary lymphocytes infiltrating the salivary and lacrimal glands during the initial stages of pSS. Additionally, elevated levels of T cell-associated cytokines have been observed in pSS patients who present with skin manifestations. These findings support the early consideration of T cell-targeted therapies, especially for patients with dermatological symptoms. Such therapies, exemplified by abatacept, have gained prominence due to their critical role in the complex pathogenesis of SS, as outlined in Table 1. Abatacept, a fusion protein consisting of the extracellular domain of human cytotoxic T-lymphocyte-associated antigen 4 and a modified Fc portion of human IgG1, is approved for rheumatoid arthritis (RA). It interferes with the CD80/86-CD28 co-stimulatory pathway, thereby inhibiting T-cell activation. Studies have shown that abatacept reduces the number of circulating follicular helper T (Tfh) cells and the expression of the T-cell surface activation marker ICOS in the peripheral blood of pSS patients. This reduction in activated Tfh cells contributes to suppressing the hyperactivity of Tfh cell-dependent B cells (Verstappen et al., 2017). Additionally, human cytotoxic T-lymphocyte-associated antigen 4 has been demonstrated to regulate CD4^+^ T cell proliferation and reduce T cell activation in SS (Manoussakis et al., 1999).

Initially, abatacept was found to lessen inflammation and enhance saliva production in SS (Meiners et al., 2014), with subsequent ROSE and ROSE II trials confirming improvements in various outcomes, including glandular, extraglandular, and systemic manifestations (Tsuboi et al., 2023). However, despite these biological effects on biomarkers and immune cells, a phase III trial in active primary SS indicated that abatacept did not achieve significant clinical efficacy compared to placebo (Baer et al., 2021; van N et al., 2020).

#### 4.2.3 Limitations and alternatives

While the targeting of T cell activation through adhesion molecule interactions has gained attention, concerns regarding adverse effects persist. For instance, alefacept, a drug designed to target T cells binding to CD2, has shown promise in achieving long-term remission in psoriasis but comes with apprehensions due to substantial T cell depletion. Similarly, efalizumab, a humanized monoclonal antibody that interferes with T cell activation, reactivation, and migration, faced safety issues in SS, leading to its discontinuation, including potential risks like progressive multifocal leukoencephalopathy.

T cell-targeted therapies have emerged as a promising avenue in SS treatment, exemplified by the potential benefits of abatacept in addressing glandular inflammation and promoting saliva production. However, the delicate balance between efficacy and safety remains a challenge, as evidenced by the limitations of alefacept and efalizumab. Given the intricate interplay between T cell dysregulation and autoimmune pathogenesis, there is a continuous need for exploring innovative therapeutic strategies that effectively modulate T cell responses while mitigating potential risks.

Lately, in a Phase II clinical trial, Dazodalibep, an antagonist of the CD40 ligand, significantly improved patients’ ESSDAI and ESSPRI scores compared to placebo. These improvements were sustained over time, demonstrating positive effects in alleviating symptoms of the disease such as dryness, fatigue, and pain (St et al., 2023). Dazodalibep emerges as a promising new therapeutic candidate for managing systemic disease activity in patients with SS. To substantiate its clinical efficacy, further extensive and large-scale clinical studies are warranted.

### 4.3 Anti-TNF-α-targeted therapies in SS

#### 4.3.1 Mechanism

TNF-α, secreted by glandular T cells, is implicated in promoting glandular epithelial apoptosis and the induction of inflammation through Fas induction (Matsumura et al., 2000). In SS patients, TNF-α is notably elevated in the acinar and ductal cells of the salivary glands (Sisto et al., 2010). In a mouse model engineered to express high levels of TNF-α, researchers observed severe inflammation in the salivary glands, along with acinar cell atrophy, fibrosis, and ductal dilation (Limaye et al., 2019). This observation is particularly significance due to the heightened production of TNF-α within glandular lesions. Inhibiting TNF-α can also suppress the production of matrix metalloproteinase-9 induced by it, thereby preventing the destruction of alveolar cells and the extracellular matrix. Therefore, mechanistically, anti-TNFα therapy should have a beneficial effect on SS (Azuma et al., 2000; Mignogna et al., 2005). However, current research indicates that this treatment has not achieved the anticipated outcomes.

#### 4.3.2 The efficacy and challenges of Anti-TNF therapies in SS

Infliximab, the first anti-TNF therapy explored in SS, initially demonstrated statistical improvement in clinical and functional parameters in a pilot trial (Steinfeld et al., 2013). However, subsequent larger RCTs failed to establish any beneficial effects (Mariette et al., 2004). Trials investigating etanercept also yielded negative outcomes (Moutsopoulos et al., 2008). Challenges such as achieving therapeutic concentrations in salivary glands and overcoming inflammation-associated fibrosis could have contributed to this treatment failure (Table 1). Therefore, anti-TNF therapies are not recommended for the treatment of SS.

#### 4.3.3 Limitations and insights from animal models

The outcomes of clinical trials partially align with insights from animal models. TNF appears to attenuate T cell autoreactivity and inflammation, and its absence leads to the accumulation of reactive CD4^+^ T cells. Emerging data from these models suggest a protective role of TNF in SS, as TNF deficiency exacerbates SS-like disease, the absence of TNF in certain models associated with inflammation within salivary glands and altered marginal B cell compartments, which might contributes to lymphoid tumors resembling MALT-L (Nocturne et al., 2021).

Anti-TNF-α-targeted therapies in SS face significant challenges, and their efficacy is questioned based on clinical trials and insights from animal models. The efficacy of anti-TNF therapies, despite their success in other autoimmune conditions, remains equivocal in the context of SS, potentially attributed to the complex and multifaceted role of TNF in the disorder’s pathogenesis (Azuma et al., 1997). The exploration of the discrepancies between the pathological mechanisms and the actual therapeutic efficacy of TNF therapy presents an interesting research direction.

### 4.4 IFN and anti-IFN-targeted therapies in SS

#### 4.4.1 Mechanism

Interferon (IFN) proteins exhibit significant immunomodulatory and antiviral properties. In the context of SS, an in-depth analysis of gene expression profiles has unveiled distinctive activation signatures associated with the IFN pathway in peripheral blood leukocytes and minor salivary gland biopsies (Båve et al., 2005; Kimoto et al., 2011; Bodewes et al., 2018). Type I interferons play a pivotal role in promoting inflammatory responses by activating peripheral blood mononuclear cells among other immune cells. Numerous studies have documented the overexpression of Type I IFN in these cells, a phenomenon known as the ‘Type I IFN signature.’ This observation is intricately linked to the onset of systemic extra-glandular manifestations, accompanied by a notable production of autoantibodies and inflammatory cytokines (Del Papa et al., 2021). Furthermore, Type I interferons are crucial in inducing B cells to produce autoantibodies, such as anti-SSA and anti-SSB, which are hallmark features of autoimmune responses in SS. IFN-γ, a canonical cytokine, induces the activation of T and NK cells and plays dual roles in immunomodulation (Zeng et al., 2022), also enhancing the class switching of immunoglobulins in B cells, thereby further promoting the production of autoantibodies.

In a recent study, the activity of Type I (IFN-I) and Type II (IFN-II) interferons in patients with SS was investigated, along with their relationship to clinical features. Patients were categorized into three groups: those with no IFN activation, those with IFN-I activation, and those with both IFN-I and IFN-II activation. The findings indicated no significant differences among the groups in terms of ESSPRI, fatigue, or dryness. However, the groups with active IFN showed higher ESSDAI scores and lower pain scores. Consequently, in future clinical trials targeting the IFN pathway, the ESSDAI score may serve as a more sensitive indicator (Bodewes et al., 2018).

#### 4.4.2 Application of IFN-α agonists in SS

IFN pathway activation signatures in SS have sparked interest in harnessing IFN-α agonists for therapeutic benefit. Early-phase trials suggest the potential for improving sicca symptoms and saliva production (Del Papa et al., 2021). A Phase III clinical trial demonstrated that low doses of oral IFN increase unstimulated salivary output. Oro-mucosal administration of IFN-α is believed to enhance saliva secretion by up-regulating aquaporin 5 transcription, without disrupting the autoimmune process (Cummins et al., 2003). However, larger double-blind, placebo-controlled RCTs involving a substantial number of patients failed to establish significant differences in oral dryness or stimulated salivary flow. Notably, an increase in unstimulated salivary flow was observed in treated groups, indicating potential therapeutic effects. Despite these findings, the clinical benefits of IFN-α agonists for SS patients remain uncertain. Thus, the IFN-α therapy is presently not recommended for treating SS.

#### 4.4.3 Limitations and anti-IFN-α mAbs

Considering IFN-α′s pro-inflammatory role, efforts have been directed towards targeting it. Two monoclonal antibodies (mAbs) directed against IFN-α, namely, rontalizumab and sifalimumab, have been explored. Rontalizumab demonstrated safety but lacked efficacy in a phase II trial (Kalunian et al., 2016). Sifalimumab also exhibited a favorable safety profile, but its efficacy in improving SS-related symptoms and markers of disease activity remained modest. Furthermore, it is worth noting that anti-IFN-α therapy has been linked with gastrointestinal adverse effects in SS (Choudhary et al., 2023). These results suggest that the development of anti-IFN-α mAbs offers an alternative approach, despite their potential, anti-IFN-α mAbs may face limitations in achieving substantial clinical benefits, which underscore the complexity of targeting IFN pathways in SS. Continued research and refinement of therapies are essential to unravel the intricate interplay of IFN and its modulation in the context of SS.

### 4.5 Therapies targeting ILs in SS

#### 4.5.1 Mechanism

Elevated levels of IL-6 observed in the serum, saliva, and tears of SS patients serve as a compelling indicator of the cytokine’s pivotal and integral role within the pathophysiological framework of SS (Ohyama et al., 1996; Grisius et al., 1997; Yoshimoto et al., 2011). IL-6 is a key factor in B cell activation, and T cell differentiation, and is associated with fatigue. Tocilizumab, a monoclonal antibody, effectively inhibits IL-6 signaling by blocking the IL-6 receptor.

#### 4.5.2 Application of tocilizumab in SS

Although tocilizumab is routinely used to treat rheumatoid arthritis, it is not yet approved for SS. However, given its ability to disrupt IL-6-mediated inflammatory processes, it presents a promising avenue for intervention in SS. While limited clinical data are available, a single case report has documented the beneficial effects of tocilizumab in a patient with neuromyelitis optica spectrum disorder complicated by SS (Komai et al., 2016). Additionally, a phase III RCT led by French investigators is comparing tocilizumab to placebo in SS patients. However, this trial failed to provide the efficacy of tocilizumab in systemic involvement and symptoms compared with placebo (Table 1) (Felten et al., 2021b).

#### 4.5.3 Limitations and future prospects

The elevation of IL-6 in SS highlights its potential as a therapeutic target. Tocilizumab, known for its efficacy in disrupting IL-6-mediated pathways, has shown promise. Although a single case report offers encouraging evidence, a large-scale Phase III study in France did not confirm tocilizumab’s effectiveness in addressing systemic involvement or alleviating symptoms in SS. Therefore, the role of targeting IL-6 in the treatment of SS remains a subject worthy of further investigation. Moreover, elucidating the broader impacts of targeting IL-6 in SS, including its potential impact on fatigue and autoimmune mechanisms, remains a topic of interest and ongoing research.

### 4.6 Therapies targeting JAK/STAT in SS

#### 4.6.1 Mechanism

The Janus kinases (JAK)-signal transducers and activators of the transcription (STAT) pathway assume a central role in autoimmunity and systemic inflammation, governing the production of inflammatory cytokines, including ILs, TNFs, granulocyte-macrophage colony-stimulating factors, and IFN-γ (Morris et al., 2018). Data concerning JAK and STAT expression within pSS-afflicted salivary glands remain sparse. However, research by Aota et al. has shed light on robust JAK1 and JAK2 expression in ductal and acinar cells of minor salivary gland biopsies from pSS patients (Aota et al., 2021). Elevated expression of STAT1 and STAT3 in minor salivary gland biopsies from pSS patients, as well as in their blood samples, has been correlated with activation triggered by a range of immune mediators, including IFN-α, IFN-γ, IL-6, IL-17, and IL-22 (Wakamatsu et al., 2006; Ciccia et al., 2012).

#### 4.6.2 Application of JAK inhibitors in SS

JAK inhibitors, approved for immune disorders and under investigation in autoimmune diseases, offer a promising therapeutic approach in rheumatology by competitively binding to ATP and modulating critical molecular and biological processes, with potential applications in pSS. In the context of the complex cytokine landscape characterizing pSS, numerous JAK inhibitors, including baricitinib, filgotinib, tofacitinib, oclacitinib, and upadacitinib, have found applications in the treatment of autoimmune diseases (Tanaka et al., 2022). In a recent pilot trial of 11 active pSS patients, the JAK1 and JAK2 inhibitor, baricitinib, showed promise in reducing immune cell infiltration and improving clinical manifestations, although controlled trials are needed for validation (Bai et al., 2022). Filgotinib, a JAK1 inhibitor, has shown potential in reducing IFN-related genes and BAFF in pSS. A clinical trial, while not meeting primary and secondary endpoints, might suggests promise for filgotinib in subgroups of pSS patients with biomarker guidance, stabilizing salivary and tear production and reducing IFN activity (Price et al., 2022). The overall safety and tolerability profile is encouraging, indicating the need for more targeted approaches in pSS clinical trials (Table 1).

#### 4.6.3 Limitations and future prospects

Notable, promising research by Renaudineau and colleagues suggests that JAK1/2 inhibitors, AG490 and ruxolitinib, may reverse specific pathways implicated in pSS pathogenesis (McCoy et al., 2022). Tofacitinib, a JAK1 and JAK3 inhibitor, has the potential to treat pSS by restoring autophagy and mitigating inflammation, particularly by targeting IL-6 expression (Barrera et al., 2021). Additionally, JAK1/2 inhibitors can counteract ROS-induced ten-eleven translocation 3 production and IFNα-mediated DNA hydroxymethylation, suggesting promise for pSS treatment (Charras et al., 2019; Charras et al., 2020).

Recent discoveries have unveiled the engagement of STAT3 in epigenetic DNA methylation and hydroxymethylation processes within pSS, impacting genes regulated by IFN-α, IFN-γ, and oxidative stress pathways (Table 1) (Charras et al., 2020). The promise of JAK-STAT inhibition in treating immune-mediated disorders, including pSS, is underscored by the intricate cytokine landscape and the key role of IFN pathways in pSS. While existing data are encouraging, further research is needed to fully grasp the implications of JAK-STAT pathway inhibition in pSS, extending beyond inflammation control to restoring salivary gland epithelium functions.

### 4.7 Unexplored potential therapies in SS

#### 4.7.1 Cytokine blockade therapies

In the landscape of SS treatment, a host of cytokines have shown promise in other autoimmune conditions, yet their potential in SS remains untapped. Cytokine blockade strategies have demonstrated efficacy in diseases like psoriasis, RA, multiple sclerosis, and inflammatory bowel disease. For instance, the blockade of IL-12 and IL-23 using briakinumab and ustekinumab has yielded success in various autoimmune disorders. Similarly, Fontolizumab, an IFN-γ blocker, is being evaluated for Crohn’s disease. IL-17 inhibition, employed in conditions such as inflammatory bowel disease, dry eye, psoriatic arthritis, and RA, presents another avenue worth exploring. Furthermore, the perturbation of IL-1, implicated in SS pathogenesis, has shown promise in ameliorating fatigue. Although these cytokine-targeting therapies bear potential for SS treatment, their direct investigation in the context of SS is yet to be undertaken.

Ongoing clinical trials are investigating various approaches to target SS (Table 1). A case report describes the significant efficacy of bortezomib, a proteasome inhibitor typically used in the treatment of multiple myeloma, in improving patient fatigue and overall quality of life (Jakez-Ocampo et al., 2015). Similarly, trials are exploring monoclonal antibodies against IL-23, including ustekinumab (Chimenti et al., 2015), briakinumab, and tildrakizumab, which are currently used in the treatment of other autoimmune diseases (Markham, 2018; Verstockt et al., 2023). Additionally, there are ongoing clinical trials aimed at targeting interferons, exemplified by NCT05383677. These trials represent continued efforts to advance our understanding and treatment of SS.

#### 4.7.2 Targeting toll-like receptors

The intricate interplay exhibited by Toll-like receptors (TLRs) in orchestrating the initiation of both innate and adaptive immune responses highlights their innovative and promising role as therapeutic targets (Zheng et al., 2010; Guerrier et al., 2012; Karlsen et al., 2017; Shimizu et al., 2019). TLR dysregulation is linked to salivary gland inflammation in SS. Inhibition of TLR-7 by IRS-954 demonstrated in a SLE murine model, could hold promise for SS intervention. Furthermore, the overexpression of TLR-9 in SS salivary glands and peripheral blood mononuclear cells is noteworthy (Zheng et al., 2010; Guerrier et al., 2012), potentially influencing aberrant B cell differentiation, and opens a path for therapeutic exploration, though its specific role remains to be elucidated.

#### 4.7.3 Chemokine-mediated lymphocyte trafficking

Chemokines, orchestrators of lymphocyte trafficking and lymphoid structure formation, bear significance in SS due to the presence of ectopic lymphoid structures in affected glandular tissues. These structures are notably characterized by the overexpression of chemokines, including CXCL13 and CXCL21 (Amft et al., 2001; Barone et al., 2005; Lee et al., 2017; Loureiro-Amigo et al., 2021). The regulation of lymphocyte behavior by these chemokines suggests their therapeutic potential. However, despite their relevance, no treatment targeting these chemokines has been investigated in SS to date.

#### 4.7.4 Exploring diverse molecular targets

Additional molecules with implications in SS manifestations and complications hold therapeutic promise. Serum fms-like tyrosine kinase 3 ligand, linked to lymphoma risk in SS, presents itself as a potential biomarker and therapeutic target (Tobón et al., 2013). Elevated CD6 expression on B and T cells in SS-affected salivary glands opens the door for itolizumab, an anti-CD6 monoclonal antibody, as an immune activation inhibitor (Alonso et al., 2010; Le Dantec et al., 2013). Furthermore, the inhibition of the lymphotoxin β receptor in a murine SS model suggests its viability as a molecular target. However, a phase II trial showed that the baminercept, a lymphotoxin β receptor IgG fusion protein, blocks lymphotoxin β receptor signaling while failing to significantly improve glandular and extraglandular disease in patients with primary SS (St Clair et al., 2018).

While numerous cytokines and molecular targets have displayed efficacy in other autoimmune conditions, their potential in SS is a largely unexplored terrain. Cytokine blockade, TLR inhibition, chemokine modulation, and exploration of diverse molecules hold promise for revolutionizing SS treatment. However, rigorous investigation, encompassing controlled trials and mechanistic studies, is essential to unravel their therapeutic potential and translate them into effective therapies for SS patients.

## 5 Advancing biologic therapies in SS: navigating complexity and embracing promise

Biologic therapies have revolutionized the landscape of SS management, providing potential treatments and advancing our understanding of immune dysregulation. Recent large-scale studies have classified patients with SS from various perspectives, including immunological patterns, clinical symptoms, and histological phenotypes. These investigations have revealed significant heterogeneity among patients with SS. Research involving extensive cases of SS, multi-omics analyses, and detailed clinical parameters has facilitated the development of personalized treatment approaches.

The intricate interplay of Th1, Th17, and B cells in persistent inflammation and autoantibody production in SS is highlighted in a review, with IL-6 emerging as a central orchestrator. Notably, T cell-targeted interventions exhibit limited efficacy, emphasizing the crucial role of autonomous B cells in driving cytokine production. Varied outcomes in B cell depletion therapies’ trials underscore the need for nuanced patient selection, especially favoring those in early stages with active extraglandular involvement.

While the horizon of biologic therapies for SS is replete with potential, the journey is not devoid of challenges. The ongoing trials investigating IL-6 and 17 targeting agents, along with agents targeting BAFF, attest to the evolving landscape of SS therapeutics. Given the intricate heterogeneity in SS patient presentations and the intricate immune responses at play, predicting individual responses to biologic therapies remains a formidable challenge.

Moving forward, nuanced approaches to patient selection for biologic therapy, targeting IL-6, IL-17, and BAFF, show promise. However, predicting individual responses to biologic therapies remains challenging, given the intricate heterogeneity in SS patient presentations. Comprehensive RCTs combining diverse therapeutic targets and patient profiles will play a pivotal role, providing evidence-based insights into the superiority of biologic therapies over conventional treatments.

In conclusion, the ongoing refinement in SS management involves navigating the complex immunological landscape, patient diversity, and a rapidly evolving therapeutic frontier. Meticulous research, strategic clinical trials, and insights from the European study collectively contribute to the promise of innovative therapies, marking a significant step towards a brighter future for SS patients.

## 6 Conclusion

The pursuit of biologic therapies holds the promise of ameliorating symptoms and thwarting the progression of SS. As scientific understanding of SS mechanisms advances and tailored therapeutic interventions are charted, judicious consideration of patient heterogeneity and empirically grounded treatments is paramount. The ongoing exploration through clinical trials and innovative research endeavors heralds a promising trajectory for individuals grappling with SS, promising improved quality of life and enhanced management of this intricate autoimmune affliction. The future of pSS treatment may involve gene, stem-cell, and tissue-engineering therapies. This review offers insights into advancing pSS management through innovative biologic interventions.

## Figures and Tables

**Table:** 

**ACPA**	Anti-citrullinated peptide antibodies
**APRIL**	a proliferation-inducing ligand
**BAFF-R**	BAFF receptor
**BCMA**	B-cell maturation antigen
**BLyS/BAFF**	B lymphocyte stimulator/B-cell activating factor
**BR3**	B-lymphocyte stimulator receptor 3
**ClinESSDAI**	Clinical ESSDAI
**CXCL**	C-X-C motif chemokine ligand
**ESSDAI**	the EULAR Sjögren’s Syndrome disease activity index
**ESSPRI**	the EULAR Sjögren’s Syndrome Patient Reported Index
**HCQ**	hydroxychloroquine
**HPA**	hypothalamic-pituitary-adrenal
**IFN**	interferon
**IL**	interleukin
**ILD**	interstitial lung disease
**ITP**	immune thrombocytopenia
**JAK**	Janus kinases
**mAbs**	monoclonal antibodies
**LELs**	lymphoepithelial lesions
**MALT-L**	mucosa-associated lymphoid tissue-lymphoma
**MCL**	mantle cell lymphoma
**MMF**	mycophenolate mofetil
**MTX**	methotrexate
**NLR**	neutrophil-to-lymphocyte ratio
**NSIP**	non-specific interstitial pneumonitis
**PGA**	patient global assessment
**PBC**	primary biliary cholangitis
**pSS**	Primary Sjögren’s Syndrome
**RA**	rheumatoid arthritis
**RCTs**	randomized controlled trials
**RF**	rheumatoid factor
**RTX**	rituximab
**SS**	Sjögren’s Syndrome
**SLE**	systemic lupus erythematosus
**STAT**	signal transducers and activators of the transcription
**TACI**	transmembrane activator and cyclophilin ligand interactor
**Tfh**	follicular helper T
**TNF**	tumour necrosis factor
**TLRs**	Toll-like receptors
**Th cell**	T helper cell
